# Abnormal Serotonin Levels During Perinatal Development Lead to Behavioral Deficits in Adulthood

**DOI:** 10.3389/fnbeh.2018.00114

**Published:** 2018-06-06

**Authors:** Relish Shah, Emmanuelle Courtiol, Francisco X. Castellanos, Catia M. Teixeira

**Affiliations:** ^1^Emotional Brain Institute, Nathan Kline Institute for Psychiatric Research, Orangeburg, NY, United States; ^2^CNRS UMR 5292 – INSERM U1028, Lyon Neuroscience Research Center, Université Lyon 1, Lyon, France; ^3^Department of Child and Adolescent Psychiatry, Hassenfeld Children’s Hospital at NYU Langone, New York, NY, United States; ^4^Division of Clinical Research, Nathan Kline Institute for Psychiatric Research, Orangeburg, NY, United States

**Keywords:** serotonin, development, SSRI, tryptophan depletion, perinatal, maternal separation

## Abstract

Serotonin (5-HT) is one of the best-studied modulatory neurotransmitters with ubiquitous presynaptic release and postsynaptic reception. 5-HT has been implicated in a wide variety of brain functions, ranging from autonomic regulation, sensory perception, feeding and motor function to emotional regulation and cognition. The role of this neuromodulator in neuropsychiatric diseases is unquestionable with important neuropsychiatric medications, e.g., most antidepressants, targeting this system. Importantly, 5-HT modulates neurodevelopment and changes in its levels during development can have life-long consequences. In this mini-review, we highlight that exposure to both low and high serotonin levels during the perinatal period can lead to behavioral deficits in adulthood. We focus on three exogenous factors that can change 5-HT levels during the critical perinatal period: dietary tryptophan depletion, exposure to serotonin-selective-reuptake-inhibitors (SSRIs) and poor early life care. We discuss the effects of each of these on behavioral deficits in adulthood.

## Introduction

Serotonin (5-hydroxytryptamine, 5-HT) is a classic and well-studied neuromodulator. In addition to being implicated in various functions ranging from autonomic regulation to emotions in the adult, 5-HT also has a key role in neurodevelopment. Indeed, in the mammalian brain, 5-HT is one of first neurotransmitters to emerge. In rodents, serotonergic neurons appear on embryonic day (E) 12 (Lauder and Bloom, 1974), and start releasing 5-HT at E13 (Lidov and Molliver, 1982a,b; Lambe et al., 2000). Levels of 5-HT peak during the first postnatal week, declining thereafter to adult levels by postnatal day (P) 15 (Hohmann et al., 1988). The expression of 5-HT receptors starts between E12 and E17 with variations and shifts across the different subtypes and regions (Waeber et al., 1994; Beique et al., 2004; Bonnin et al., 2006; for review: Booij et al., 2015).

In humans, 5-HT neurons start to appear when the embryo is 5 weeks old and proliferate until gestational week 10 (Sundstrom et al., 1993; Levallois et al., 1997; Gingrich et al., 2017). Levels of 5-HT increase during the first 2 years of age and slowly decline to reach adult levels by age 5 (Sodhi and Sanders-Bush, 2004).

Importantly, 5-HT is involved in neural crest stem cell migration and proliferation (Vichier-Guerre et al., 2017). Notably, 5-HT can shape neuronal microcircuitry by acting on Reelin secretion, a protein involved in neuronal migration and positioning during development (Chameau et al., 2009). 5-HT is also critical in cell survival, growth and differentiation (Lavdas et al., 1997; Fricker et al., 2005; Khozhai and Otellin, 2012) as well as synaptogenesis (Khozhai and Otellin, 2012). For reviews see (Gaspar et al., 2003; Daubert and Condron, 2010).

**FIGURE 1:**
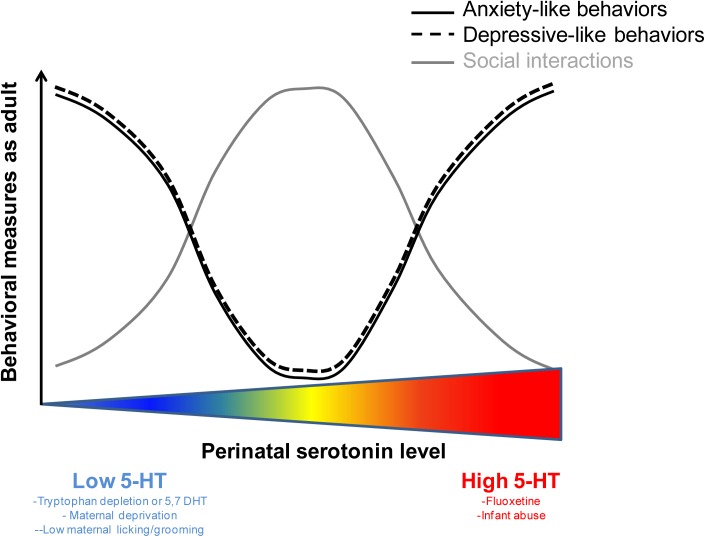
The relation between perinatal levels of serotonin and behavioral performance as an adult is non-linear. U-shapped and inversed U-shape curves illustrating the optimal levels of perinatal serotonin sustaining normal behavioral performance in adulthood. Deviation from these optimal levels (either above or below) can lead to behavioral deficits in the adult.

Plasticity during the perinatal period is essential for the developing brain to adapt to a changing environment but opens a window where external factors can derail neuronal circuits and lead to maladaptive behaviors. This perinatal time window is a critical period when serotonergic activity can shape the development of neuronal circuitry and specifically emotional neurocircuitry (Lauder, 1990; Brummelte et al., 2017). In addition to genetic variants in key serotonergic genes [e.g., SERT, MAO; see (Murphy et al., 2008; Suri et al., 2014)], several exogenous factors change 5-HT levels during development: stress (Papaioannou et al., 2002), physical abuse (Raineki et al., 2015; Rincon-Cortes et al., 2015), food intake (Zhang et al., 2006; Serfaty et al., 2008; Zoratto et al., 2013), pharmaceutical and recreational drugs (Xu et al., 2004; Suri et al., 2014) as well as maternal inflammation (Goeden et al., 2016; St-Pierre et al., 2016) and maternal separation (Shannon et al., 2005). Importantly, manipulations that either increase or decrease 5-HT levels during development have been shown to lead to behavioral deficits in the adult (**Table 1**). Together, these studies suggest that optimal levels of 5-HT must be maintained during development and that any deviations from these optimal levels, in either direction, can lead to long-lasting behavioral deficits (**Figure 1**). In this mini-review, we focus on three distinct exogenous factors, diet, pharmacological exposure and early-life care, which affect serotonin levels during the perinatal period and have behavioral consequences in adulthood.

**Table 1:** Manipulations of the serotonergic system during development lead to behavioral deficits observed during adulthood: Examples of diet, pharmacological agents and early-life maternal care effects.

Treatment		Effect on 5-HT signaling	Species and Sex	Age of exposure	Behavior change (measured in adulthood)	Reference
Diet-induced	Tryptophan depletion	Decrease	Mice - 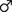	P0-P8	Low break point in progressive ratio Deficits in approach-avoidance conflict paradigms	Zoratto et al., 2013
	Tryptophan depletion	Decrease	Rat - 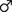	P1-P28	Higher immobility in the forced-swim-test Less time in open arms of the elevated plus maze	Zhang et al., 2006
Pharmacological: Neurotoxin or tryptophan hydroxylase inhibitor	5,7 DHT	Decrease	Rat - 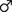	P3	Reduced locomotor activity Reduced social interaction Increased ultrasonic vocalization in fear-conditioning	Rok-Bujko et al., 2012
	PCPA	Decrease	Rat - 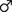	P10-20	Delayed extinction in interchangeable mazes task Increased number of errors in eight-arm radial maze	Mazer et al., 1997
Pharmacological: SSRI	Fluoxetine	Increase	Rat - 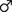 – 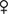	E6- E20	Transient delay in motor development Improvements in the water maze and passive avoidance tests	Bairy et al., 2007
	Fluoxetine	Increase	Rat - 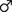 – 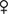	E11-birth	Increased anxiety-like behavior in the novelty-suppressed feeding test, the footshock-induced conditioned place aversion test and in the elevated plus maze	Olivier et al., 2011
	Fluoxetine	Increase	Mice - 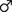 – 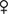	P2/P4- P21	Decreased exploratory behavior Increased anxiety-like behavior in the novelty-suppressed-feeding paradigm Impaired performance in active avoidance	Ansorge et al., 2004; Yu et al., 2014
	Fluoxetine	Increase	Mice - 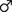 – 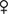	P2- P21	Reduced aggression	Yu et al., 2014
	Fluoxetine	Increase	Rat - 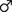	10 days during infancy	Increased immobility in the forced-swim-test	Rincon-Cortes et al., 2015
	Fluoxetine	Increase	Mice - 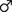 – 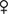	P2-P11	Exploratory deficits Increased latency to feed in the novelty-suppressed feeding test Increased escape latency in the shock-avoidance test Decreased sucrose consumption Increased immobility time in the forced-swim-test	Rebello et al., 2014
Early-life maternal care Maternal care	Low maternal licking/ grooming	Decrease	Rat - 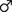 – 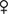	Early-life	Impaired stress response	Francis et al., 1999; Weaver et al., 2004; Hellstrom et al., 2012
	Infant abuse (Odor-shock conditioning)	Increase	Rat - 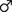	P8-P12	Deficits in social behavior Increased immobility in the forced-swim-test	Rincon-Cortes et al., 2015
	Maternal deprivation (maternal separation)	Increase 5-HT2 receptor function	Rat - 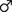	P2-P14	Increased 5-HT2R agonist-induced head-shake behavior Increased anxiety-like behavior in open field test and the elevated plus maze (blocked by 5-HT2R antagonist)	Benekareddy et al., 2010, 2011
	Maternal deprivation (nursery reared)	Lower 5-HIAA levels	Monkey - 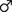	From 24h post-birth	Increased alcohol consumption	Huggins et al., 2012
	Maternal deprivation (maternal rejection)	Lower 5-HIAA levels	Monkey - 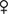	From birth	Increased scratching (indicator of anxiety-like behaviors) Increased solitary play	Maestripieri et al., 2006

*Postnatal day (P) – Embryonic day (E); 5,7-dihydroxytryptamine (5,7 DHT) and parachlorophenylalanine (PCPA). 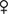 = Female; 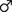 = Male.*

## Diet-Induced Disruption of 5-Ht Synthesis: Tryptophan Depletion

Tryptophan is a 5-HT precursor and reducing its availability substantially reduces 5-HT synthesis (Biggio et al., 1974; Moja et al., 1989; Fadda et al., 2000). Since tryptophan cannot be internally synthesized, diets low in tryptophan have been used to decrease 5-HT synthesis.

For ethical reasons, tryptophan depletion studies in humans have only been performed in adults. Such diets have been found to induce depressive symptoms in healthy individuals with a family history of depression and lead to relapse in depressed patients who were previously treated with selective-serotonin-reuptake-inhibitors (SSRIs) (Benkelfat et al., 1994; Delgado et al., 1999; Klaassen et al., 1999; Quintin et al., 2001). For an in-depth review of the behavioral consequences of acute, adult, tryptophan depletion in humans see (Faulkner and Deakin, 2014).

In rodents, only a few studies have examined the long-term effects of low tryptophan diet during early development. Early studies (Segall and Timiras, 1976; Segall et al., 1983) found that female rats starting a diet low in tryptophan at an early developmental age (weaning age-3 weeks) displayed a delay in growth, ovarian function and sexual maturation. Those effects were suggested to be related to low 5-HT in the pituitary-pineal glands. More recently, administration of a low tryptophan diet to lactating dams was found to reduce maternal care. Importantly, this intervention also induced anxiety-like and anhedonia-related behaviors in the adult offspring (Zoratto et al., 2013). Moreover, early life administration of a maize-based diet, containing approximately 20% of tryptophan of regular diets led to increased immobility in the forced-swim-test, reduced time in the open arms of the elevated plus maze, decreased neurogenesis and abnormal dendritic development in the dentate gyrus at 4 weeks of age (Serfaty et al., 2008).

Tryptophan depletion and pharmacologically disrupting 5-HT synthesis using a selective neurotoxin, 5,7-dihydroxytryptamine (5,7 DHT), greatly differ given that the latter leads to permanent neuronal loss. However, the long-term effects of a low-tryptophan diet can be compared to the effects of disrupting 5-HT synthesis with 5,7 DHT. Indeed, administering this neurotoxin during development also disrupts behavior in the adult. 5,7 DHT administration at P3 in rats led to reduced locomotor activity, attenuated social interaction and increased ultrasonic vocalization during fear-conditioning (Rok-Bujko et al., 2012). In addition, depletion of serotonin (P10-20) using the tryptophan hydroxylase inhibitor (parachlorophenylalanine, PCPA) decreases dendritic density (microtubule-associated protein) in hippocampus up to P62 and produces deficits in spatial learning and delayed extinction interpreted as a deficit in normal response inhibition (Mazer et al., 1997). The effects in adulthood of a transient 5-HT disruption during development highlight the importance of precise regulation of serotonergic levels during critical developmental periods.

In summary, reducing 5-HT synthesis during perinatal life, through tryptophan depletion or 5,7 DHT administration, leads to maladaptive behavioral phenotypes including depressive-like and anxiety-like symptoms as well as defective social interactions.

## Pharmacological Modifications of Perinatal 5-Ht Levels: Exposure to SSRIs

SSRIs are the most prescribed treatments for depression due to their safety and mild-to-moderate adverse effects, with several being approved for use in children and adolescents (Henry et al., 2012). Pregnancy is a risk factor for depression and SSRIs are taken by a substantial proportion of women (∼ 10%) during pregnancy (Cooper et al., 2007; Huybrechts et al., 2013) possibly exposing the fetus to increased 5-HT levels. SSRIs cross the placenta barrier (Hendrick et al., 2003; Rampono et al., 2009; Sit et al., 2011) and can also be passed to the baby via breast milk (Kristensen et al., 1999). Notably, due to the possibly devastating effects of depression, including potential suicide, discontinuation of antidepressant treatment during pregnancy is often problematic.

Because of the altricial nature of rodents, the 3rd trimester of pregnancy in humans corresponds in terms of development of the serotonergic system to the first postnatal weeks in rodents (Suri et al., 2014). This is the period during which the serotonergic system’s development is at its peak (Suri et al., 2014), which presumably makes it more vulnerable to disruption. To test the influence of SSRIs during the perinatal stage in rodents, two approaches have been taken. These are exposure to SSRIs *in utero* or during the early-postnatal period (Kepser and Homberg, 2015). In-depth reviews on the effects of developmental exposure to antidepressant medications can be found in (Olivier et al., 2013; Kepser and Homberg, 2015).

The offspring of pregnant rats exposed to the prototypic SSRI, fluoxetine, given orally at 12 mg/kg, from E6 to E20 showed a decrease in birth weight, a transient delay in motor development and improvements in performance in the water maze and passive avoidance tests tested post-weaning (Bairy et al., 2007). In Olivier et al. (2011), pregnant rats were injected daily with 12 mg/kg of fluoxetine from E11 until birth. Adult offspring exhibited increased anxiety-like behaviors in the novelty-suppressed feeding test, the footshock-induced conditioned place aversion test and in the elevated plus maze (Olivier et al., 2011). In Rincon-Cortes et al. (2015), daily administration of 8 mg/kg of fluoxetine to rat pups for 10 days during infancy produced depressive-like behaviors in the adult, i.e., increased immobility in the forced-swim-test (Rincon-Cortes et al., 2015). Postnatal exposure to 10 mg/kg fluoxetine from P2 to P21 in mice led to decreased exploratory behavior, increased anxiety-like behavior in the novelty-suppressed feeding paradigm and impaired active avoidance performance at adulthood (Ansorge et al., 2004; Yu et al., 2014). Furthermore, this treatment reduced aggression when the mice were tested as adults (Yu et al., 2014). Interestingly, the detrimental effects of fluoxetine were restricted to a critical period from P2 to P11 (Rebello et al., 2014). Indeed, fluoxetine administration during this period, but not after, was sufficient to cause exploratory deficits, increased latency to feed in the novelty-suppressed feeding test and increases in escape latency in the shock-avoidance test (Rebello et al., 2014). Furthermore, these mice showed depression-like behaviors with decreased sucrose consumption and increased immobility time in the forced-swim-test (Rebello et al., 2014). Interestingly, these deleterious effects were specific to SSRIs. Postnatal treatment with fluoxetine, clomipramine and citalopram all produced emotional behavioral deficits in mice while administration of the norepinephrine transporter inhibitor, desipramine, did not (Ansorge et al., 2008).

Concerning the possible neurobiological mechanisms underlying the lasting effects of fluoxetine administered during the critical P2-P11 period, modulation of the serotonergic system during this period has been shown to lead to changes in prefrontal cortex pyramidal neuron morphology and reduced excitability (Rebello et al., 2014). 5-HT2 receptors are major modulators of cortical serotonergic signaling. 5-HT2 activation results in neuronal depolarization and increases excitatory postsynaptic currents (Celada et al., 2013b). Interestingly, blocking 5-HT2 signaling concomitantly with fluoxetine administration in pups prevents the behavioral deficits observed with fluoxetine administration alone (Sarkar et al., 2014), suggesting that 5-HT2 receptors may mediate serotonergic effects during this period.

In humans, although fewer studies exist than in rodents, there is also some evidence that perinatal exposure to SSRIs can lead to behavioral deficits. In a study using Finnish national registry data, the incidence of depression by age 14.9 years in offspring exposed prenatally to SSRIs was 8.2% (*N* = 15,729) compared to 1.9% (*N* = 9,651) in the control group with psychiatric disorders but no medication (Malm et al., 2016). In addition to possible effects in mood-related development, exposure to SSRIs during development has been associated with an increased incidence of autism in children (Boukhris et al., 2015), which is congruent with the observation of hyperserotonemia in close to one third of autistic children (Hranilovic et al., 2009). An in depth review of the effects of perinatal SSRI administration in humans and animal models can be found in (Olivier et al., 2013).

In summary, increasing 5-HT signaling during a critical period of perinatal life, via exposure to SSRIs, has been shown to lead to emotional deficits in adulthood (e.g., increased anxiety-like and depressive-like behaviors).

## Early-Life Maternal Care: Relationship With the Serotonergic System and Long-Term Behavioral Consequences

Emotional deficits related to poor quality of care during early-life are a major social issue. The prevalence of child maltreatment is alarmingly high (Finkelhor et al., 2009) with important societal consequences (U.S. Department of Health and Human Services, 2010). Indeed, early life experience is critical for life-long mental health as it is a period of high plasticity (Chaudhury et al., 2016). One of the major environmental inputs during early life is the caregiver. Importantly, in both humans and rodents, fear and stress responses (Routh et al., 1978; Hellstrom et al., 2012; Rincon-Cortes et al., 2015) as well as changes in mood (Vetulani, 2013) and drug consumption (Vetulani, 2013) are regulated by the quality of maternal care during early life.

Numerous studies have demonstrated the link between maternal care, serotonergic signaling and later vulnerability to mental health issues. Seminal work by Meaney and colleagues describe an increase in basal glucocorticoid levels, as well as in the secretion of glucocorticoids in response to stress in rats that were less groomed and licked by their mothers (Hellstrom et al., 2012). This effect was found to be mediated through the serotonergic system (Mitchell et al., 1990, 1992; Laplante et al., 2002; Hellstrom et al., 2012). Indeed, Hellstrom et al. (2012) suggest that 5-HT, resulting from licking/grooming, acts via the 5-HT7 receptor to activate a signaling cascade that in turn leads to the activation of transcription factors such as NGFI-A (nerve growth factor-inducible factor A) leading to demethylation of the glucocorticoid receptor promoter (Laplante et al., 2002; Hellstrom et al., 2012).

In monkeys, early-life maternal separation reduced serotonin transporter binding (Ichise et al., 2006) and lowered the levels of cerebrospinal 5-HIAA (a 5-HT metabolite) (Shannon et al., 2005; Maestripieri et al., 2006, 2009). Importantly, naturalistic maternal rejection, associated with lower cerebrospinal fluid 5-HIAA levels, was associated with anxiety-like behaviors later in life (Maestripieri et al., 2006). Furthermore, 5-HIAA in the putamen and 5-HIAA/5-HT ratio measures of 5-HT turnover in the hippocampus (and at trend levels in caudate, Substantia nigra and putamen) were significantly lower in nursery reared compared to mother-reared animals; ethanol drinking was also higher in nursery reared monkeys (Huggins et al., 2012).

In rodents, maternal separation has been shown to affect the tissue level of both 5-HT and 5-HIAA in different brain structures. Arborelius and Eklund (2007) found that maternal separation (P1-P13) modified the tissue levels of 5-HT and 5-HIAA in the dorsal raphe nucleus (both increased during long maternal separation) and in the amygdala (decreased during brief maternal separation) of adult female rats. Moreover, the 5-HIAA/5-HT ratio in the rat prefrontal cortex was decreased by maternal separation (Xue et al., 2013; Gonzalez-Mariscal and Melo, 2017). Analysis of the metabolite/monoamine turnover ratio is a useful estimator of serotonergic activity. An increase in this ratio indicates increased serotonergic turnover usually associated with higher rates of 5-HT release into the synapse (Shannon et al., 1986).

Maternal separation also affects 5-HT receptor expression in adulthood. Indeed, adult animals subjected to maternal separation display modifications of 5-HT1A receptor mRNA expression with a decrease in the dorsal raphe nucleus and an increase in the amygdala (Bravo et al., 2014). Postsynaptic 5-HT1A receptor activation in corticolimbic areas appears anxiolytic, while high density of presynaptic 5-HT1A receptors increases susceptibility to mood disorders and activation of these presynaptic receptors can reduce raphe signaling through a negative-feedback system (Celada et al., 2013a). However, an increase in post-synaptic 5-HT1A in the amygdala can also produce anxiogenic behaviors (Gonzalez et al., 1996). The behavioral endpoint of the two distinct regional alterations in 5-HT1A receptor levels may be dictated by the differential density of this receptor in each structure.

In addition, maternal separation also leads to a dysregulation of 5-HT2 receptor function in the prefrontal cortex (Benekareddy et al., 2010). Behaviorally, 5-HT2A agonist-induced head-twitch responses, a model for psychosis and hallucinations in rodents, are potentiated by maternal separation (Benekareddy et al., 2010). Moreover, maternal separation increases anxiety-like behaviors (measured in the open field and the elevated-plus-maze test) and 5-HT2 receptor expression. These effects have been shown to be blocked by treatment with the 5-HT2 antagonist, ketanserin (Benekareddy et al., 2011). Developmental physical abuse as modeled by paired odor-shock conditioning, has also been shown to increase 5-HT levels and lead to deficits in social behavior and increased immobility in the forced swim-test in the adult (Rincon-Cortes et al., 2015). Importantly, both deficits can be rescued by increasing amygdala 5-HT and suppressing corticosterone (Rincon-Cortes et al., 2015), an effect that may be paralleled to the aforementioned work of Hellstrom et al. (2012).

In summary, early life maternal separation or abuse induce modifications of serotonergic signaling (for example increased 5-HT level or increased 5-HT2 receptor expression) that are correlated with later-on maladaptive behaviors such as deficits in social interactions and anxiety-like and depressive-like behaviors in adulthood.

## Discussion

In this mini-review, we highlighted the striking and enduring effects on adult behavior of both low and high 5-HT levels during the perinatal period. However, many questions remain. Notably, how do both low and high 5-HT levels during development lead to similar behavioral deficits? One hypothesis is that both can lead to a reduction in serotonergic signaling in the adult. For instance, tryptophan depletion may directly impair the development of the serotonergic system (Mazer et al., 1997) while excessive serotonergic signaling may reduce serotonergic activity/development through negative feedback. Serotonergic innervation peaks during the perinatal period. Perhaps excessive serotonin during this period falsely signals a sufficiency of serotonergic growth leading to stunted development. In fact, maternal inflammation was recently found to lead to increased levels of 5-HT in the fetal brain which subsequently inhibits serotonergic development (Goeden et al., 2016). Furthermore, the firing rate of serotonergic cells in the dorsal raphe of postnatal fluoxetine-treated animals decreases dramatically during adulthood (Teissier et al., 2015). Future experiments, taking advantage of the ability to apply opto- and chemo-genetics tools in neonatal models (Bitzenhofer et al., 2017) can further help dissect how both high and low 5-HT levels can disrupt the development of the serotonergic system and affect behavior in the adult.

This review highlights the critical period when any deviations of 5-HT levels can lead to later maladaptive behavior. For example, Rebello et al. (2014) demonstrated that vulnerability to fluoxetine is restricted to P2-P11. This apparent critical period can be explained by (1) the timeline of brain developmental processes such as proliferation of neurons and glia, migration, apoptosis, and synaptogenesis encompassing both the embryonic stages as well as the first postnatal weeks in rats (Rice and Barone, 2000) and (2) the ontogeny of serotonergic system development [for review see (Booij et al., 2015)]. Indeed, while the serotonergic system continues to mature after birth, there are also critical shifts of receptor expression and activity during this period. For example, from P6 to P19, pyramidal neurons change from being depolarized to being hyperpolarized by 5-HT through different 5-HT receptor subtypes (Beique et al., 2004).

Another question that remains unsettled related to whether the vulnerability to abnormal 5-HT during the perinatal period leading to maladaptive behaviors in adulthood differs in males and females. Indeed, as shown in **Table 1**, except for the early exposure to SSRIs, most prior studies have only used male animals. While sex differences in animal models of vulnerability to psychiatric disorder and early life stress are increasingly demonstrated (Dalla et al., 2010; Reincke and Hanganu-Opatz, 2017) and a sexual dimorphism in serotonergic system activity in adults is known (Carlsson and Carlsson, 1988; Haleem et al., 1990), the systematic study of the effects of 5-HT level variations during the perinatal period in both sexes remains a high priority.

In this review, we chose to present examples of three exogenous factors supporting the hypothesis of a non-linear relationship between serotonin-levels during early-life and adult behavior. In addition to the models on which we focused, other exogenous factors and genetic models/variants support the hypothesis that both high and low levels of 5-HT during development lead to behavioral deficits, and more specifically to emotional deficits. MAO-A/B-KO mice, which have increased monoamine levels, show a significantly decreased distance traveled in the open-field, and a reduction in the time spent in open-arms in the elevated-plus-maze (Chen et al., 2004). Similarly, constitutive SERT-KO mice, with elevated 5-HT levels, present anxiety-like and depression-like behaviors such as reduced locomotor activity in the open-field, increased latency-to-feed in the novelty-suppressed-feeding test and social-interaction deficits (Lira et al., 2003; Ansorge et al., 2004; Kalueff et al., 2007). Hyposerotonergic function in Lmx1b-raphe specific-KO mice, leads to increased freezing in the fear conditioning test and impaired water-maze memory retrieval (Dai et al., 2008). Constitutively reduced serotonergic levels in a model of SERT-overexpression resulted in reduced anxiety-like phenotypes in the elevated-plus-maze and in the hyponeophagia tests (Jennings et al., 2006). Although the latter study suggests that hyposerotonergic function may be protective of anxiety-like behaviors, other studies using mutant mice with severe 5-HT ablations point to the inverse. While Pet1-KO mice, which only retain residual serotonergic innervation, exhibited reduced anxiety-like behaviors and increased fear responses in one study (Kiyasova et al., 2011), Pet1-KO mice showed increased anxiety-like behaviors and increased aggression in another (Hendricks et al., 2003). Furthermore, Tph2- R441H-KI mice, in which 5-HT brain levels are approximately 80% of wildtype, exhibit increased immobility in the tail suspension test and increased latency to cross to the light chamber in the Dark-Light test (Beaulieu et al., 2008).

Finally, these studies raise the urgent question of whether the behavioral deficits caused by altered 5-HT levels during development can be reversed in adulthood. In Lmx1b-raphe specific-KO mice, plasticity deficits were rescued with a bath application of 5-HT (Dai et al., 2008). Interestingly, Tph2- R441H-KI behavioral deficits could be rescued by administration of a GSK3-β inhibitor (Beaulieu et al., 2008). However, it is striking that adult behavior is impaired in both the SERT-KO mouse (lifelong impaired serotonergic transporter) and the temporally restricted administration of a transporter blocker during perinatal-life (fluoxetine from P4-P21) (Ansorge et al., 2004). This demonstrates that increased serotonergic tone in the adult SERT-KO is not sufficient to rescue the deficits caused by increased serotonergic signaling during development.

## Conclusion

High perinatal plasticity allows the brain to adapt to its environment but also marks a period of increased vulnerability. The regulation of serotonin levels during development is extremely important for typical brain development. Both low and high levels of serotonergic signaling seem to be detrimental, leading to multiple behavioral deficits. Many exogenous factors can affect 5-HT levels during development. Future studies need to address the boundary conditions and underlying mechanisms of optimal 5-HT levels during the critical perinatal period to optimize long-term behavioral and emotional outcomes.

## Author Contributions

All authors listed have made a substantial, direct and intellectual contribution to the work, and approved it for publication.

## Conflict of Interest Statement

The authors declare that the research was conducted in the absence of any commercial or financial relationships that could be construed as a potential conflict of interest.
